# Post-Chemotherapy Changes and Agreement of CT-Derived Body Composition at L3 and T12 in Older Patients with Metastatic Colorectal Cancer: Associations with Nutritional Indices and Outcomes

**DOI:** 10.3390/nu18071090

**Published:** 2026-03-28

**Authors:** Anıl Yıldız, Melin Aydan Ahmed, Nihan Nizam Eren, Abdulmunir Azizy, Selay Artan, Simay Çokgezer, Bedirhan Ulufer, Ozan Deniz Aygörmez, Gündüz Karaoğlan, Şirin Zelal Şahin Tırnova, Gulistan Bahat, Mustafa Durmaz, İnci Kızıldağ Yırgın, Senem Karabulut, Burak Sakar, Mehmet Akif Karan, Didem Taştekin

**Affiliations:** 1 Department of Medical Oncology, Başaksehir Çam ve Sakura City Hospital, 34480 İstanbul, Türkiye; gunduzkaraoglan@gmail.com; 2 Department of Medical Oncology, Istanbul University Oncology Institute, 34093 İstanbul, Türkiye; drmelinahmed@gmail.com (M.A.A.); munir.azizy@gmail.com (A.A.); selayartan@gmail.com (S.A.); simaycokgezer@gmail.com (S.Ç.); bedirhanulufer@hotmail.com (B.U.); ozanaygormez@gmail.com (O.D.A.); drsenemkarabulut@gmail.com (S.K.); buraksakar@gmail.com (B.S.); didem_doktor@hotmail.com (D.T.); 3 Department of Medical Oncology, Bağcılar Research and Education Hospital, 34212 İstanbul, Türkiye; nihannizam@gmail.com; 4 Department of Internal Medicine, Division of Geriatrics, Istanbul Medical Faculty, Istanbul University, 34320 İstanbul, Türkiye; zelalsahin86@gmail.com (Ş.Z.Ş.T.); gbahatozturk@yahoo.com (G.B.); m.akifkaran@gmail.com (M.A.K.); 5 Department of Radiology, Istanbul University Oncology Institute, 34093 İstanbul, Türkiyeinci.kizldag@gmail.com (İ.K.Y.)

**Keywords:** metastatic colorectal cancer, sarcopenia, skeletal muscle index, intramuscular adipose tissue, nutritional index, chemotherapy toxicity, survival

## Abstract

Background: Age- and cancer-related sarcopenia and malnutrition are common in older patients with colorectal cancer (CRC) and may negatively influence treatment tolerance and prognosis. However, the comparative prognostic value of post-chemotherapy changes in CT-based body composition parameters at the third lumbar vertebra (L3) and the twelfth thoracic vertebra (T12) levels, and their associations with nutritional indices, remain unclear. This study aimed to examine and compare the prognostic relevance of post-chemotherapy body composition changes at L3 and T12 and to assess their relationship with nutritional indices in older patients with metastatic CRC (mCRC). Methods: This retrospective study included 87 older patients with mCRC. Baseline and ~3-month follow-up CT scans were analyzed at L3 and T12 using 3D Slicer to quantify skeletal muscle index (SMI), subcutaneous adipose tissue index (SATI), visceral adipose tissue index (VATI), visceral-to-subcutaneous fat ratio (VSR), and intramuscular adipose tissue index (IMATI). Changes (Δ) in CT-derived body composition after chemotherapy were calculated as percentage change using ((follow-up − baseline)/baseline) × 100. Prognostic Nutritional Index (PNI) and Geriatric Nutritional Index (GNRI), which are established nutritional assessment tools, were calculated from baseline laboratory/anthropometric data. Agreement between T12 and L3 was assessed, and associations with grade ≥ 3 toxicity, progression-free survival (PFS), and overall survival (OS) were evaluated using multivariable models and ROC analyses. Results: Mean age was 69.0 ± 4.5 years (59 male/28 female), and 26.4% developed grade ≥ 3 adverse events. Over 3 months, mean SMI declined significantly at both L3 (46.7 ± 8.8 → 42.8 ± 9.8 cm^2^/m^2^) and T12 (34.6 ± 8.2 → 31.6 ± 8.1 cm^2^/m^2^) (*p* < 0.001 for both), accompanied by decreases in VATI and VSR; T12-IMATI increased significantly. Baseline PNI showed a weak positive correlation with L3-SMI (r = 0.302, *p* = 0.033), whereas GNRI showed moderate correlations with SMI at L3 (r = 0.502, *p* < 0.001) and T12 (r = 0.317, *p* = 0.025) and was associated with longitudinal changes in muscle metrics. T12-SMI consistently yielded lower values than L3-SMI, and agreement varied by compartment (best for SATI; weakest for VSR). Lower GNRI and greater L3-SMI loss were independently associated with grade ≥ 3 toxicity; ΔL3-SMI showed the highest discrimination (AUC = 0.79, 95% CI = 0.69–0.87, *p* < 0.001; cut-off >5.1% loss). All patients progressed (median PFS 7.6 months); mortality was 82.8% (median follow-up: 25 months). In multivariable analysis, PFS, CRP, GNRI, and ΔL3-SMI remained independently associated with OS. ΔL3-SMI provided the strongest mortality discrimination (AUC = 0.85, 95% CI = 0.74–0.94, *p* < 0.001; cut-off >10.4% loss), while ΔIMATI was also informative (AUC = 0.71, 95% CI = 0.59–0.82, *p* = 0.023). Conclusions: In older patients with mCRC, early post-chemotherapy skeletal muscle loss—particularly at the L3 level—showed the strongest prognostic association with severe toxicity and mortality. GNRI provided complementary prognostic information as a marker of baseline immunonutritional reserve. Although T12-derived measurements were correlated with L3-derived values, systematic bias suggests that they should not be interpreted interchangeably for longitudinal risk stratification.

## 1. Introduction

Colorectal cancer (CRC) is predominantly diagnosed in older adults, and the expanding older population means an increasing share of patients begin treatment with reduced physiological reserve, multimorbidity, and heightened vulnerability to functional decline [1]. Beyond tumor stage and molecular features, host factors—especially low muscle mass, systemic inflammation, and nutritional depletion—can shape chemotherapy tolerance, postoperative recovery, and long-term survival [2]. In recent CRC meta-analytic evidence, computed tomography (CT)-defined loss of muscle mass (typically quantified on routine staging CT) is common and consistently associated with worse overall and disease-free survival [3]. Cancer-associated metabolic and inflammatory signaling can further accelerate muscle wasting in an older patient during cancer treatment, potentially amplifying frailty and limiting the ability to sustain planned oncologic therapy [4]. Malnutrition and frailty are highlighted as prevalent in older CRC populations and linked to poorer outcomes, supporting the need for pragmatic markers that capture nutritional and immune reserve [5].

CT-based body composition assessment has emerged as a valuable and objective approach for quantifying treatment-related changes in skeletal muscle and adipose tissue compartments. Prognostic evidence in CRC has relied on the third lumbar vertebra (L3)-based CT metrics, encompassing not only skeletal muscle index (SMI) but also multiple adipose tissue parameters, with muscle quantity, muscle quality, and fat distribution carrying prognostic relevance [6,7]. Longitudinal analyses indicate that early treatment-related muscle loss—rather than weight loss—may provide meaningful prognostic information, as demonstrated by studies showing that post-chemotherapy or post-diagnosis skeletal muscle decline is associated with worse survival outcomes [8,9].

Although L3 remains the reference standard for CT-based body composition analysis, evaluation of alternative vertebral landmarks remains clinically relevant. Thoracic imaging is routinely relevant in colorectal cancer as part of metastatic assessment, particularly for pulmonary disease evaluation, and the twelfth thoracic vertebra (T12) is a thoracoabdominal transition level that still permits quantification of skeletal muscle and adipose tissue compartments. In addition, alternative landmarks may be useful when the L3 slice is not optimally assessable because of heterogeneous scan coverage, limited field of view, local artifacts, anatomical distortion, postoperative change, or segmentation difficulty. Therefore, comparing T12 with L3 is important not only to assess cross-level correlation, but also to determine whether T12 may serve as a complementary landmark and whether these two levels should or should not be interpreted interchangeably for longitudinal prognostic assessment.

Comparative studies evaluating T12 and L3 have suggested significant cross-level correlations and have shown that low post-treatment SMI at either level is associated with poorer survival, supporting the biological plausibility of T12 as an alternative landmark [10,11]. Measurements obtained at different vertebral levels differ in terms of visible muscle groups, inclusion of abdominal wall musculature, and susceptibility to respiratory motion and thoracic anatomical variability, factors that may influence segmentation reliability and measurement behavior [12]. Although several studies have reported significant correlations between thoracic and lumbar muscle indices, systematic bias and level-specific performance differences have also been observed, suggesting that these landmarks may not be fully interchangeable when assessing body composition changes [10,13]. In parallel, nutritional indices such as the Prognostic Nutritional Index (PNI) and the Geriatric Nutritional Risk Index (GNRI) have been shown to have independent prognostic value in CRC and have been linked to low muscle mass, reinforcing the concept that laboratory-based nutritional risk and imaging-derived muscle depletion are related but non-redundant domains [14,15]. Despite this, it remains unclear whether baseline nutritional indices can predict post-chemotherapy changes in CT-derived muscle and adipose tissue parameters, and whether such associations differ between L3 and T12 assessments.

In light of the above considerations, whether post-chemotherapy changes in CT-derived body composition assessed at different vertebral levels provide comparable prognostic information in older CRC patients remains unclear. In addition, although immunonutritional indices such as the PNI and the GNRI are established prognostic markers, their relationship with longitudinal trajectories of CT-derived muscle and adipose tissue parameters following chemotherapy has not been well defined. Because body composition and nutritional status reflect underlying physiological reserve, they may influence both treatment tolerance and long-term outcomes. Therefore, evaluating chemotherapy-related toxicity and survival outcomes alongside nutritional status and CT-derived body composition parameters may provide a more comprehensive understanding of vulnerability and prognosis in older patients with metastatic CRC (mCRC). Accordingly, the aim of the present study was to compare the prognostic significance of post-chemotherapy changes in CT-based body composition parameters measured at the L3 and T12 vertebral levels in older mCRC patients and to examine their association with immunonutritional indices.

## 2. Materials and Methods

This retrospective cohort study included older patients (≥65 years) diagnosed with mCRC who were treated and followed at the Istanbul University Oncology Institute between January 2013 and December 2024. The study adhered to the ethical regulations and principles specified in the Declaration of Helsinki and received approval from the Ethical Committee of Istanbul University Oncology Institute (Date: 11 June 2024, Decision No: 2602581). The requirement for obtaining informed consent was exempted by the Ethics Committee due to the retrospective design of the study.

### 2.1. Study Population

Over the study period, 116 mCRC patients were retrospectively screened. Patients aged ≥65 years with histologically confirmed colorectal carcinoma, an Eastern Cooperative Oncology Group (ECOG) performance status 0–2, and metastatic disease measurable according to the Response Evaluation Criteria in Solid Tumors (RECIST), who received at least one line of systemic chemotherapy after the diagnosis of stage IV disease, were eligible for inclusion. Additional inclusion criteria were normal liver, bone marrow, and renal function; availability of contrast-enhanced CT scans containing evaluable axial images at the L3 and T12 vertebral levels at baseline (prior to chemotherapy) and approximately 3 months after initiation of chemotherapy; and complete blood count and biochemical parameters obtained within 1 week prior to systemic treatment. Patients were excluded if CT images were unavailable or of insufficient quality for body composition analysis, if they had another primary malignancy during the study period, autoimmune or hematologic diseases, clinical evidence of active infection, or if they were lost to follow-up or had incomplete clinical or laboratory data. The final study population comprised 87 patients.

### 2.2. Data Collection and Definitions

Clinical and demographic variables were extracted from electronic medical records. Collected variables typically included: age at diagnosis, sex, height and body weight (for index normalization and GNRI calculation), comorbidity burden (e.g., Charlson Comorbidity Index if available), ECOG performance status, tumor location (colon/rectum), stage at diagnosis, use of oral nutritional support, treatment intent and line of chemotherapy, and receipt of surgery and/or radiotherapy where relevant. Treatment-related information (regimen class, number of cycles in the first 3 months, dose modifications, treatment delays, and discontinuation) was recorded, given their potential relationship with nutritional status and body composition change.

The use of oral nutrition support (ONS) during chemotherapy was recorded retrospectively from patient records. ONS was defined as prescription of commercially available oral nutritional supplements initiated at any time during treatment. Given the potential impact of nutritional interventions on treatment tolerance and outcomes, ONS use was included as a clinically relevant variable in the analysis.

Tumors were staged according to the American Joint Committee on Cancer (AJCC) TNM classification (7th edition) [16]. All patients received either oxaliplatin (e.g., FOLFOX or CAPOX), irinotecan (e.g., FOLFIRI), or capecitabine-based regimens as part of their treatment protocol. Dose delay was defined as a delay of at least 5 days in treatment due to chemotherapy-related side effects, and dose reduction was defined as a reduction in chemotherapy dose by at least 10% in at least one chemotherapy cycle due to chemotherapy-related side effects. Survival status and follow-up dates were obtained from hospital records systems.

### 2.3. Laboratory Measurements

Baseline laboratory parameters were retrospectively collected within one week prior to the initiation of chemotherapy. Routine blood parameters were analyzed using a Beckman Coulter DxH 800 hematology analyzer (Beckman Coulter, Miami, FL, USA). Leukocyte counts were obtained via impedance; C-reactive protein levels through immunoturbidimetry; and albumin through the bromocresol green method. PNI was calculated using the formula (10 × albumin [g/dL]) + (0.005 × lymphocyte count [/mm^3^]); patients were classified as low-PNI (<46) or high-PNI (≥46) [17]. GNRI = 1.489 × serum albumin (g/L) + 41.7 × body weight/ideal body weight [18].

### 2.4. CT-Based Body Composition Analysis

Baseline (pre-treatment) and approximately 3-month post-chemotherapy follow-up CT scans were retrospectively retrieved from the institutional image archiving and communication system (PACS). The follow-up scans were identified manually from routine clinical imaging records, and the CT examination closest to 3 months after initiation of chemotherapy was selected for body composition analysis at the T12 and L3 vertebral levels. All segmentations were performed using the 3D Slicer software (3D Slicer, version 5.6.2, open-source image computing platform). For each time point, one axial CT slice was selected at the T12 and L3 levels using a standardized approach to ensure consistent vertebral localization across patients and between scans.

**Figure 1:**
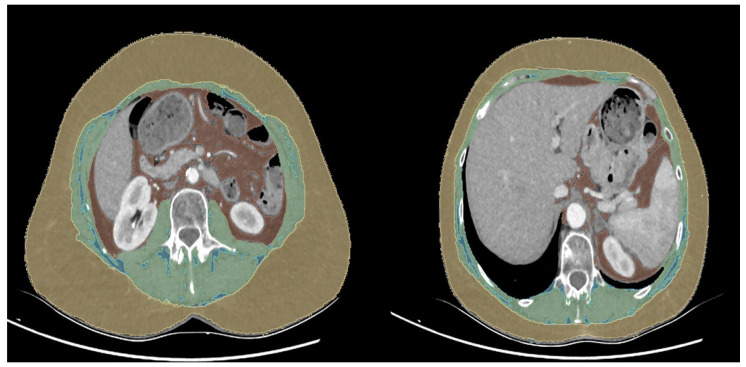
Computed tomography-based body composition analysis at the third lumbar vertebra (L3, **left panel**) and the twelfth thoracic vertebra (T12, **right panel**) levels.

At each level, cross-sectional areas were quantified for skeletal muscle, visceral adipose tissue (VAT), and subcutaneous adipose tissue (SAT). At L3, skeletal muscle segmentation included all muscles visible on the slice (psoas, erector spinae, quadratus lumborum, transversus abdominis, internal and external obliques, and rectus abdominis), consistent with established oncologic body composition methodology. At T12, the skeletal muscle compartment comprised the total visible skeletal musculature at that level (predominantly paraspinal and trunk muscles, including abdominal wall musculature when present), segmented as a single muscle compartment for index calculation (Figure 1).

To evaluate measurement reproducibility, CT segmentations were independently reviewed by two radiologists (I.K.Y and M.D.) who were blinded to all clinical characteristics, laboratory results (including PNI and GNRI), treatment-related toxicity, and survival outcomes. A predefined random subset of patients (e.g., n = 15, including baseline and follow-up scans) was re-segmented by the second reader to assess inter-observer agreement. For intra-observer variability, the primary reader repeated segmentation of the same subset after a washout period of at least two weeks. Final tissue areas and derived indices were calculated according to the predefined thresholds and anatomical rules. Intraclass correlation coefficients (ICCs) were calculated to evaluate both intra-observer and inter-observer consistency, with ICC values ranging between 0.87 and 0.93, indicating excellent reproducibility [19].

Tissue segmentation was performed using standard Hounsfield unit (HU) thresholds with manual correction when necessary: −29 to +150 HU for skeletal muscle, −150 to −50 HU for VAT, and −190 to −30 HU for SAT. Intramuscular adipose tissue (IMAT) was quantified within the segmented muscle compartment using the adipose tissue attenuation range (−190 to −30 HU). All measurements were calculated as cross-sectional area [20]. Indices were calculated by normalizing tissue areas to height squared: SMI = SMA/height^2^, VATI = VAT area/height^2^, SATI = SAT area/height^2^, and IMATI = IMAT area/height^2^ (cm^2^/m^2^). The VAT-to-SAT ratio (VSR) was defined as VAT area divided by SAT area. Post-chemotherapy changes (Δ) in CT-derived body composition parameters were expressed as percentage change, calculated as the difference between follow-up and baseline measurements divided by the baseline value and multiplied by 100.

Categorical definitions were applied in accordance with the previous literature: sarcopenia was defined as an L3 SMI ≤ 38.5 cm^2^/m^2^ [21,22], and visceral obesity as VATI ≥ 100 cm^2^ [23]. All measurements were performed by experienced radiologists (I.K.Y and M.D.) to ensure consistency and reduce observer bias.

### 2.5. Outcomes

The primary outcomes of the study were the occurrence of grade ≥ 3 chemotherapy-related adverse events and overall survival (OS). Secondary outcomes included the agreement between CT-derived body composition parameters measured at the L3 and T12 vertebral levels, as well as their associations with nutritional indices (PNI and GNRI). These endpoints were analyzed together because CT-derived body composition parameters and nutritional indices reflect host physiological reserve, which may influence both treatment tolerance and survival outcomes in older patients with mCRC.

Treatment-related toxicities during chemotherapy were recorded and graded according to institutional standards, with severe toxicity defined as grade ≥ 3 adverse events documented in the medical records by the treating oncologist. Progression-free survival (PFS) was defined as the time from initiation of chemotherapy to radiologically confirmed disease progression or death from any cause, whichever occurred first. OS was defined as the time from initiation of chemotherapy to death from any cause. Patients without an event at the time of data cutoff were censored at the date of last follow-up.

### 2.6. Statistical Analysis

All statistical procedures were conducted using SPSS version 24.0 (IBM Corp., Armonk, NY, USA) and R version 4.3.0 (R Foundation for Statistical Computing, Vienna, Austria). The distribution of continuous variables was examined with the Kolmogorov–Smirnov test. Continuous data are reported as mean ± standard deviation (SD) when normally distributed, and as median with interquartile range (IQR) when the distribution was abnormal; categorical data are presented as counts and percentages. Comparisons between groups were made with Student’s *t*-test for normally distributed variables and the Mann–Whitney U test for skewed variables, whereas categorical variables were analyzed using the Chi-square test or Fisher’s exact test, as appropriate. Correlation analyses were performed using Pearson’s correlation for normally distributed variables and Spearman’s rank correlation for non-normally distributed variables. Correlation coefficient of <0.10 were evaluated as negligible correlation, 0.10–0.39 as weak correlation, 0.40–0.69 as moderate correlation, 0.70–0.89 as strong correlation, and 0.90–1.00 as an almost perfect, very strong correlation [24]. Paired comparisons of CT-based body composition parameters before and after chemotherapy were performed using the paired Student’s *t*-test for normally distributed variables and the Wilcoxon signed-rank test for non-normally distributed variables. Between-group comparisons of changes (Δ) in CT-derived body composition after chemotherapy were evaluated using mixed model for repeated measures (MMRM) analysis.

Agreement between T12-derived measurements and the reference standard L3 measurements was evaluated using Bland–Altman analysis, with L3 considered the gold standard. Mean bias (T12 − L3) and 95% limits of agreement (LoA; bias ± 1.96 standard deviations) were calculated separately for baseline and post-treatment scans. To further quantify agreement, intraclass correlation coefficients (ICC) were calculated using a two-way mixed-effects model with absolute agreement and single measurements [ICC(A,1)]. ICC values were interpreted as poor (<0.5), moderate (0.5–0.75), good (0.75–0.90), or excellent (>0.90). In addition, linear calibration models were fitted for each parameter to derive equations of the form: L3 = *a* + *b* × T12.

To identify potential predictors of grade ≥ 3 adverse events, variable selection was performed using least absolute shrinkage and selection operator (LASSO) penalized logistic regression analysis. In the LASSO models, a logistic regression with an L1 penalty was fitted, and the regularization parameter [log10(C)] was tuned by 5-fold stratified cross-validation using the area under (AUC) the receiver operating characteristic (ROC) curve analysis as the optimization criterion. The 95% confidence interval of the AUC was estimated using 1000 bootstrap resamples. Trajectories of standardized regression coefficients were plotted across the range of log10(C) values, and predictors with non-zero coefficients at the selected penalty level were carried forward to subsequent multivariable analyses [25]. The variables retained after variable selection were entered into multivariable logistic regression model (Backward:Wald method) to determine independent predictors of grade ≥ 3 adverse events. Age, sex, BMI, comorbidity burden, site of metastasis, surgical treatment, lymphovascular invasion, perineural invasion, chemotherapy regimen, and number of cycles were considered confounders and were entered into the multivariable logistic regression model. Results were expressed as adjusted odds ratios (ORs) with corresponding 95% confidence intervals (CIs).

Cox proportional hazards regression was used to estimate hazard ratios (HRs) and 95% CIs. The proportional hazards assumption was assessed using log-minus-log survival plots and Schoenfeld residual inspection. Univariate Cox models were first fitted for each parameter. Variables with *p* < 0.05 in the univariate analysis were included in the multivariable Cox regression analysis (Backward:Wald method). Age, sex, BMI, comorbidity burden, site of metastasis, surgical treatment, lymphovascular invasion, perineural invasion, chemotherapy regimen, and number of cycles were considered confounders and were entered into the multivariable Cox regression model.

The diagnostic performance of nutritional and CT-based body composition parameters in predicting study outcomes was evaluated using ROC analysis. Optimal cutoff values were derived using the Youden index, and differences between AUCs of independent predictors were compared with the DeLong test [26].

A two-tailed *p*-value < 0.05 was considered statistically significant.

## 3. Results

### 3.1. Patient Characteristics

A total of 87 older patients diagnosed with mCRC were included. The mean age was 69.0 ± 4.5 years, and 67.8% of the patients were male. At baseline, median BMI was 26.1 ± 4.6 kg/m^2^, while the mean PNI and GNRI were 44.2 ± 7.7 and 93.3 ± 6.4, respectively. Common comorbidities included hypertension (29.9%) and diabetes mellitus (17.2%), while the median CCI was 9. Most patients had ECOG performance status 0–1 (94.3%). Metastatic involvement was most frequently observed in the lymph node (89.7%) and liver (58.6%). All patients received systemic chemotherapy, with oxaliplatin- or irinotecan-based regimens being the most commonly administered. Grade ≥ 3 adverse events occurred in 26.4% of patients. The most frequent grade ≥ 3 adverse events were neutropenia in 10 patients (11.5%), followed by neuropathy in 7 patients (8.0%) and thrombocytopenia in 7 patients (8.0%). During follow-up, disease progression occurred in all patients, with a median PFS of 7.6 months. The median follow-up period was 25 months, and the mortality rate was 82.8% (Supplementary Table S1).

Baseline and post-chemotherapy changes in body composition parameters are summarized in Table 1. At the L3 level, SMI decreased significantly (*p* < 0.001), accompanied by significant reductions in VATI and VSR (both *p* < 0.001), while SATI showed no significant change (*p* = 0.856). IMATI demonstrated a significant increasing trend (*p* = 0.034). At the T12 level, SMI, VATI, and VSR also decreased significantly (*p* < 0.001, *p* = 0.001, and *p* < 0.001, respectively) and IMATI increased significantly (*p* = 0.020); no significant change was observed in SATI (*p* = 0.535). Baseline PNI showed a significant but weak positive correlation with baseline L3-SMI (r = 0.302, *p* = 0.033). In contrast, baseline GNRI showed a moderate positive correlation with SMI at both the L3 (r = 0.502, *p* < 0.001) and T12 (r = 0.317, *p* = 0.025) vertebral levels, while exhibiting a weak negative correlation with IMATI (r = −0.344, *p* = 0.016 for L3; r = −0.309, *p* = 0.035 for T12). Neither baseline PNI nor GNRI showed significant correlations with the other body composition parameters (Table 1).

Given that GNRI showed the strongest association among baseline nutritional indices, we further evaluated its ability to predict CT-based body composition measures using robust regression models. GNRI was an independent predictor of pre-chemotherapy SMI at both the L3 and T12 vertebral levels, whereas it was not independently associated with the remaining baseline body composition parameters. Regarding post-chemotherapy changes, GNRI exhibited a broadly similar pattern; however, it additionally emerged as an independent predictor of changes in IMATI (Supplementary Table S2).

### 3.2. Agreement Between T12- and L3-Derived Body Composition Parameters

**Figure 2:**
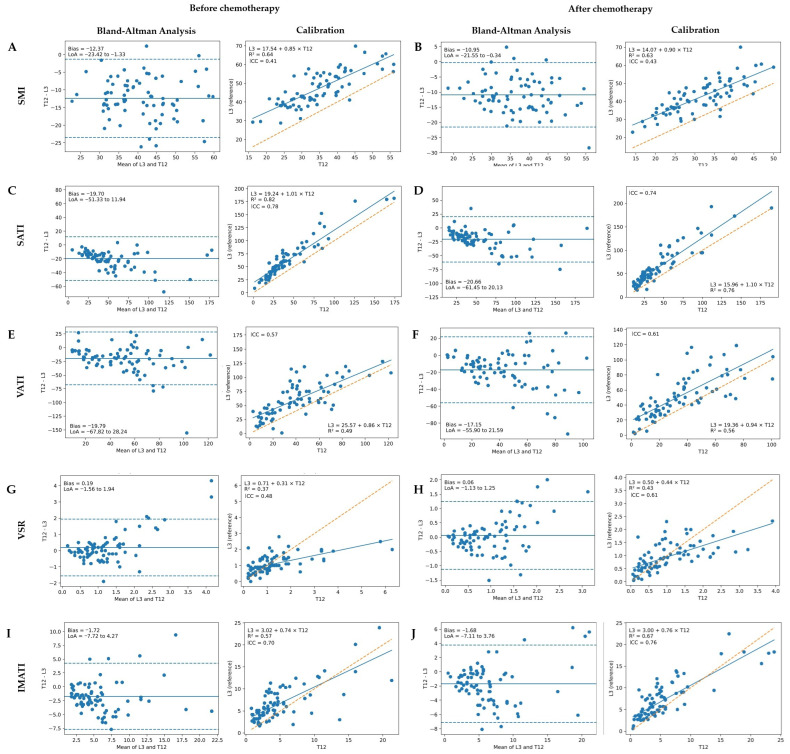
Agreement between the third lumbar vertebra (L3)-derived and the twelfth thoracic vertebra (T12)-derived body composition parameters before and after chemotherapy. Panels (**A**,**C**,**E**,**G**,**I**) present the analyses before chemotherapy, whereas panels (**B**,**D**,**F**,**H**,**J**) present the corresponding analyses after chemotherapy. (**A**,**B**): Skeletal muscle index (SMI); (**C**,**D**): Subcutaneous adipose tissue index (SATI); (**E**,**F**): Visceral adipose tissue index (VATI); (**G**,**H**): Visceral-to-subcutaneous fat ratio (VSR); (**I**,**J**): Intramuscular adipose tissue index (IMATI). For each parameter, the left panel (**A**,**C**,**E**,**G**,**I**) shows the Bland–Altman analysis, and the right panel (**B**,**D**,**F**,**H**,**J**) shows the calibration plot. In the Bland–Altman plots, the *y*-axis represents the difference (T12 − L3) and the *x*-axis represents the mean of the two measurements. The solid horizontal line indicates the mean bias, and the dashed lines indicate the 95% limits of agreement (bias ± 1.96 SD). The calibration plots display the relationship between T12 and L3 values together with the identity line and the fitted linear calibration model (L3 = a + b × T12). Intraclass correlation coefficients (ICC) are shown as a measure of relative agreement between T12- and L3-derived measurements.

At baseline, Bland–Altman analyses demonstrated systematic differences between T12- and L3-derived measurements across all evaluated parameters (Figure 2A,C,E,G,I). T12-derived SMI values were consistently lower than L3-derived SMI, as indicated by a negative mean bias and stable limits of agreement, suggesting a systematic offset between vertebral levels (Figure 2A). SATI demonstrated the most favorable agreement with L3 measurements, characterized by comparatively narrower limits of agreement and supportive ICC values, indicating good relative reliability despite the presence of small systematic differences (Figure 2C). VATI showed moderate agreement, with wider limits of agreement and moderate ICC values, reflecting increased variability across vertebral levels (Figure 2E). VSR exhibited weaker agreement, as evidenced by broader limits of agreement and lower ICC values, indicating limited concordance between T12- and L3-derived ratios (Figure 2G). IMATI demonstrated acceptable agreement at baseline, with interpretable Bland–Altman limits and moderate ICC values (Figure 2I).

Following chemotherapy, a similar overall pattern of agreement was observed (Figure 2B,D,F,H,J). T12-derived SMI remained lower than L3-derived SMI, with a bias magnitude comparable to baseline, indicating the persistence of systematic differences after treatment (Figure 2B). SATI again demonstrated favorable agreement with L3 measurements, supported by consistent Bland–Altman characteristics and good relative reliability as reflected by ICC values (Figure 2D). VATI maintained moderate agreement in the post-treatment setting, with limits of agreement and ICC values comparable to baseline findings (Figure 2F). In contrast, VSR showed relatively lower agreement after chemotherapy, indicating increased variability in ratio-based measures (Figure 2H). IMATI maintained acceptable agreement across time points, with stable Bland–Altman characteristics and moderate ICC values (Figure 2J).

### 3.3. Parameters Associated with Grade ≥ 3 Adverse Events

Patients who developed grade ≥ 3 adverse events were more likely to receive ONS than those without severe toxicity (43.5% vs. 21.9%, *p* = 0.047). Other demographic and clinical characteristics were comparable between patients with and without grade ≥ 3 adverse events (Supplementary Table S3). Patients who developed grade ≥ 3 adverse events had significantly lower serum albumin levels compared with those without severe toxicity (3.3 ± 0.4 vs. 3.7 ± 0.4, *p* < 0.001). Similarly, baseline PNI was lower in patients with grade ≥ 3 adverse events (40.4 ± 5.7 vs. 45.6 ± 8.0, *p* = 0.006), and GNRI was also lower in this group (89.2 ± 5.1 vs. 94.7 ± 6.2, *p* < 0.001). Other laboratory parameters did not differ significantly between the two groups (*p* > 0.05 for all). Regarding CT-based body composition parameters, baseline L3-SMI was lower in patients with grade ≥ 3 adverse events than those without severe toxicity (42.0 ± 7.2 vs. 46.5 ± 8.5, *p* = 0.033). A similar pattern was observed for chemotherapy L3-SMI levels (43.6 ± 8.7 vs. 36.4 ± 8.3, *p* < 0.001). Other CT-based body composition parameters were comparable between patients with and without grade ≥ 3 adverse events (Supplementary Table S4).

In both patients with and without grade ≥ 3 adverse events, significant post-chemotherapy reductions in SMI, VATI, and VSR were observed at both the L3 and T12 vertebral levels. However, the magnitude of these changes at the L3 level was more pronounced in patients who developed grade 3 adverse events (Δ*p* < 0.05). At the T12 level, only changes in SMI and IMATI differed significantly between groups (Δ*p* < 0.001 for SMI; Δ*p* = 0.038 for IMATI). While IMATI levels did not change significantly in patients without grade ≥ 3 adverse events (*p* = 0.116), patients with grade ≥ 3 adverse events exhibited a significant increase in IMATI at both L3 and T12 vertebral levels (*p* = 0.046) (Table 2).

**Figure 3:**
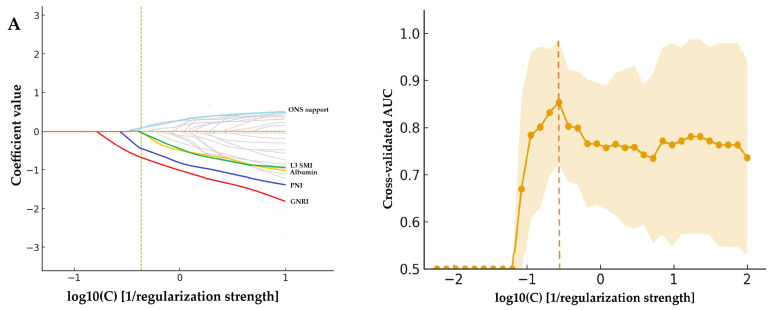

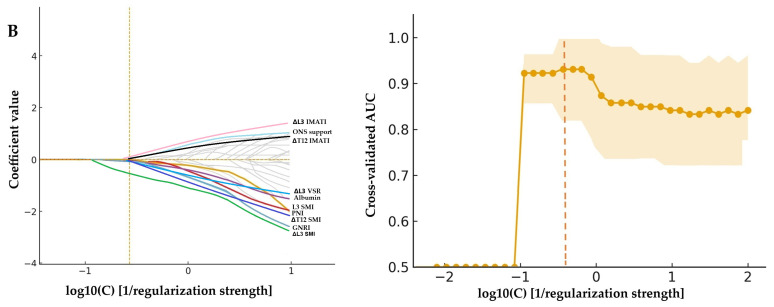
LASSO regression models for the prediction of grade ≥ 3 adverse events. (**A**) Baseline model incorporating baseline clinical, laboratory, and computed tomography-derived body composition parameters. (**B**) Delta model integrating post-chemotherapy changes (Δ) in computed tomography-based body composition parameters together with baseline nutritional indices and laboratory markers. Each curve represents the standardized coefficient of an individual variable plotted against log10(C), where C denotes the inverse of the regularization strength. As regularization decreases (rightward along the *x*-axis), additional variables enter the model with non-zero coefficients. The vertical dashed lines indicate the optimal penalization levels: log10(C) = −0.37 for the baseline model (AUC = 0.85, 95% CI = 0.74–0.95) and log10(C) = −0.57 for the delta model (AUC = 0.92, 95% CI = 0.83–0.99), both selected via 5-fold stratified cross-validation and 1000 bootstrap resampling for the 95% CI of the AUC. At these penalization levels, only variables with non-zero coefficients were retained as informative predictors. Abbreviations: AUC, area under the receiver operating characteristic curve; CI, confidence interval; ONS, oral nutritional support; SMI, skeletal muscle index; IMATI, intramuscular adipose tissue index; VSR, visceral-to-subcutaneous fat ratio; PNI, prognostic nutritional index; GNRI, geriatric nutritional risk index; Δ, post-chemotherapy change.

To identify key parameters associated with grade ≥ 3 adverse events and to reduce model dimensionality, we constructed two separate LASSO regression models. This approach enabled the shrinkage of irrelevant coefficients toward zero while retaining the most informative demographic, clinical, laboratory, and CT-based variables. The first model (baseline model) incorporated baseline clinical, laboratory, and CT-derived parameters. The coefficient path plot illustrates the shrinkage behavior of standardized coefficients across varying regularization strengths, identifying PNI, GNRI, serum albumin, and L3-SMI as the most informative baseline predictors, while ONS demonstrated a positive coefficient (Figure 3A; optimal log10(C) = −0.37). The second model (delta model) integrated post-chemotherapy changes (Δ) in key CT-based body composition parameters, including ΔL3-SMI, together with baseline nutritional indices (PNI, GNRI) and laboratory markers. In this model, ΔL3-SMI, PNI, GNRI, and albumin emerged as dominant contributors, indicating that dynamic changes in skeletal muscle mass provided incremental predictive value beyond baseline measurements alone (Figure 3B; optimal log10(C) = −0.57). Cross-validated AUC analysis demonstrated robust discriminatory performance for both models (AUC = 0.85; 95% CI 0.74–0.95 for the baseline model and AUC = 0.92; 95% CI 0.83–0.99 for the delta model). Notably, the delta model achieved a higher and more stable AUC across a range of regularization strengths, suggesting improved prediction of severe toxicity when longitudinal body composition changes were incorporated (Figure 3).

Given the higher AUC observed in the delta LASSO model, the variables retained in this model (ONS, albumin, PNI, GNRI, L3 SMI, ΔL3 SMI, ΔL3 VSR, ΔL3 IMATI, ΔT12 SMI, and ΔT12 IMATI) were further evaluated for their association with grade ≥ 3 adverse events using univariable logistic regression analysis (Table 3). In the multivariable logistic regression model including these variables, lower GNRI (OR = 0.86, 95% CI: 0.78–0.94, *p* = 0.002) and greater post-chemotherapy decline in ΔL3-SMI (OR = 0.87, 95% CI = 0.81–0.94, *p* = 0.001) emerged as independent predictors of grade ≥ 3 adverse events after adjustment for age, sex, BMI, comorbidity burden, site of metastasis, surgical history, lymphovascular invasion, perineural invasion, chemotherapy regimen, and number of cycles. Accordingly, each one-unit decrease in GNRI was associated with a 1.16-fold (1/OR 0.86) increase in the odds of severe toxicity, while each one-unit greater decline in ΔL3-SMI was associated with a 1.15-fold increase (1/OR 0.87) in the odds of grade ≥ 3 adverse events (Table 3).

**Figure 4:**
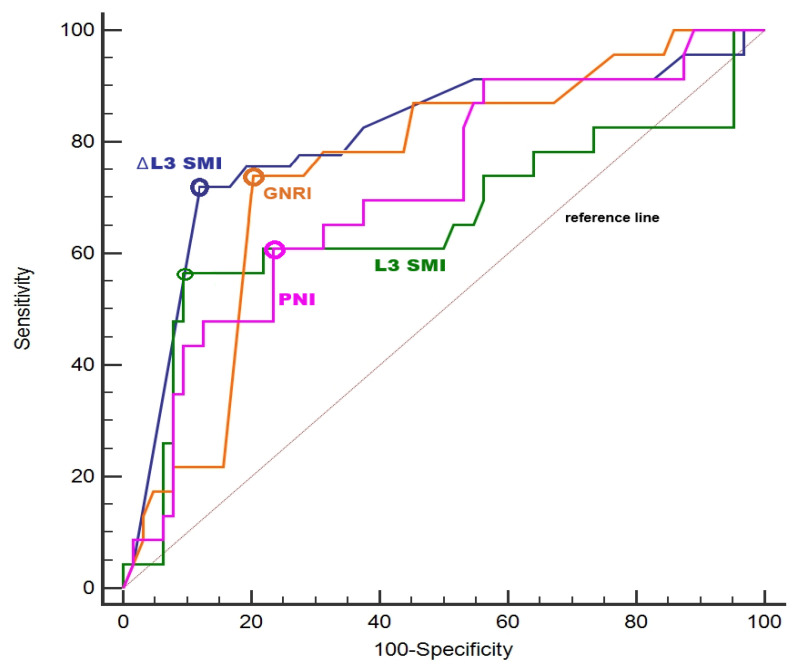
Receiver operating characteristic (ROC) curve analysis of ΔL3-SMI, GNRI, PNI, and baseline L3-SMI for predicting grade ≥ 3 adverse events. Colored circles indicate the optimal thresholds determined by maximizing Youden’s index for each ROC curve. Abbreviations: GNRI, geriatric nutritional risk index; L3, the third lumbar vertebra; ROC, receiver operating characteristic; SMI, skeletal muscle index; PNI, prognostic nutritional index.

ROC analysis showed that ΔL3-SMI had the highest discriminative ability for grade ≥ 3 adverse events (AUC = 0.79, 95% CI = 0.69–0.87, *p* < 0.001), with a cut-off value of >5.1% reduction (sensitivity 72.3%, specificity 87.9%). GNRI (AUC = 0.74, 95% CI = 0.64–0.83, *p* < 0.001; cut-off <87.8) and PNI (AUC = 0.71, 95% CI = 0.61–0.80, *p* < 0.001; cut-off <40.8) demonstrated moderate discrimination, whereas baseline L3-SMI showed lower discriminative performance (AUC = 0.65, 95% CI = 0.54–0.75, *p* = 0.038) (Figure 4).

### 3.4. Parameters Associated with Mortality

Patients with ECOG 2 were associated with an increased risk of mortality compared with those with ECOG 0–1 (HR = 2.62; 95% CI = 1.04–6.64; *p* = 0.042). Patients who underwent surgical intervention had a lower risk of mortality compared with patients who did not undergo surgery (HR = 0.59; 95% CI = 0.36–0.97; *p* = 0.036). In addition, a lower number of chemotherapy cycles was associated with a reduced risk of mortality (HR = 0.61, 95% = CI 0.38–0.97; *p* = 0.039). Longer PFS was also associated with reduced mortality risk (HR = 0.95; 95% CI = 0.90–1.00; *p* = 0.038) (Supplementary Table S5).

Among laboratory findings, lower serum albumin levels were associated with increased mortality risk (HR = 0.39; 95% CI = 0.21–0.73; *p* = 0.003). Similarly, higher CRP levels were associated with mortality (HR = 1.01; 95% CI = 1.00–1.01; *p* = 0.002). Both lower PNI (HR = 0.97; 95% CI = 0.94–0.99; *p* = 0.013) and GNRI (HR = 0.96; 95% CI = 0.93–0.99; *p* < 0.001) were associated with a higher risk of mortality. Regarding CT-based body composition parameters, baseline lower L3-SMI was associated with increased mortality risk (HR = 0.96; 95% CI = 0.94–0.99; *p* < 0.001). After chemotherapy, lower L3-SMI levels remained associated with a higher risk of mortality (HR = 0.97; 95% CI = 0.94–0.99; *p* < 0.001). No significant associations with mortality were observed for other CT-based body composition parameters at either vertebral level (Supplementary Table S6).

CT-based body composition changes at the L3 and T12 vertebral levels according to survival status are summarized in Table 4. Among surviving patients, no significant post-chemotherapy changes were observed in L3-SMI or IMATI, whereas significant reductions were noted in L3-VATI and L3-VSR (*p* < 0.05). At the T12 level, surviving patients showed modest but significant decreases in SMI and VSR, while SATI, VATI, and IMATI remained unchanged. In contrast, deceased patients demonstrated pronounced post-chemotherapy deterioration in body composition. At both L3 and T12 levels, SMI, VATI, and VSR decreased significantly, accompanied by a significant increase in IMATI (*p* < 0.05 for all). Between-group comparisons of changes (Δ*p*) revealed that declines in SMI and increases in IMATI at both vertebral levels were significantly greater in deceased patients compared with survivors (Table 4).

Variables associated with mortality in univariable analysis are summarized in Table 5, with detailed results presented in Supplementary Tables S5 and S6. In the multivariable Cox regression analysis, PFS (HR = 0.94, 95% CI: 0.90–0.98; *p* = 0.036), CRP (HR = 1.08, 95% CI: 1.02–1.14; *p* = 0.012), GNRI (HR = 0.96, 95% CI: 0.93–0.99; *p* = 0.024), and ΔL3-SMI (HR = 0.97, 95% CI: 0.95–0.99; *p* = 0.007) remained independently associated with mortality after adjustment for age, gender, body mass index, comorbidity burden, site of metastasis, surgical operation, lymphovascular invasion, perineural invasion, chemotherapy regime and number of cycles. Independent of other variables, each 1-month decrease in PFS decreased mortality risk by 1.06-fold (1/HR 0.94). Similarly, each 1-unit increase in CRP increased mortality risk by 1.08-fold, while each 1-unit decrease in GNRI and each 1% decrease in ΔL3-SMI increased mortality risk by 1.04-fold (1/HR 0.96) and 1.03-fold (1/HR 0.97), respectively (Table 5).

ROC analysis showed that baseline nutritional indices demonstrated moderate discriminative performance, with PNI (AUC = 0.70, *p* = 0.020) and GNRI (AUC = 0.76, *p* < 0.001) being significantly associated with mortality. Among pre-chemotherapy CT parameters, L3-SMI showed moderate performance (AUC = 0.72, *p* < 0.001). Post-chemotherapy changes exhibited higher discriminative performance, particularly ΔL3-SMI, which showed high performance for mortality prediction (AUC = 0.85, *p* < 0.001). In addition, ΔL3-IMATI (AUC = 0.71, *p* = 0.023), ΔT12-SMI (AUC = 0.70, *p* = 0.028), and ΔT12-IMATI (AUC = 0.68, *p* = 0.032) demonstrated moderate discriminative performance (Supplementary Table S7).

**Figure 5:**
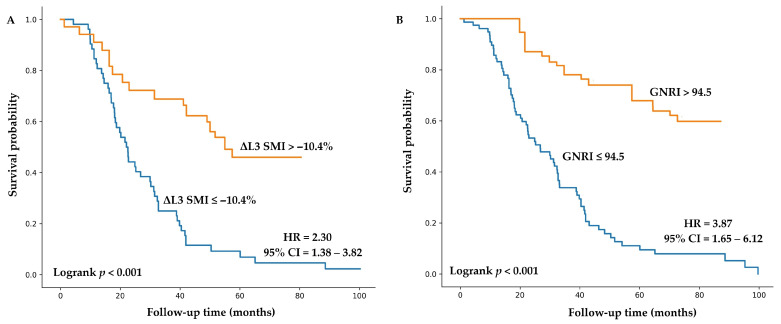
Kaplan–Meier survival curves according to post-chemotherapy change in L3-SMI (**A**) and GNRI (**B**) using predefined cut-off values. Abbreviations: CI, confidence interval; GNRI, geriatric nutritional risk index; L3, the third lumbar vertebra; SMI, skeletal muscle index; HR, hazard ratio.

Kaplan–Meier survival analysis showed that patients with GNRI ≤ 94.5 had a 3.87-fold higher risk of mortality compared with those with GNRI > 94.5 (HR = 3.87, 95% CI = 1.65–6.12; *p* < 0.001). Likewise, patients with a ≥10.4% reduction in ΔL3-SMI exhibited a 2.30-fold increased risk of mortality compared with those with lesser muscle loss (HR = 2.30, 95% CI: 1.38–3.82; *p* < 0.001) (Figure 5).

## 4. Discussion

To the best of our knowledge, this study is among the first to comprehensively investigate the association between CT-based body composition changes at the L3 and T12 vertebral levels and nutritional indices in older patients with mCRC. In this cohort, following chemotherapy, we observed decreases in SMI and concomitant increases in IMATI at both the L3 and T12 vertebral levels. Importantly, early muscle loss—particularly at L3—carried the strongest prognostic and clinical signal, outperforming baseline CT metrics and T12 change measures for outcome discrimination. Furthermore, this study suggests that, although thoracic measurements may provide useful information when abdominal levels are unavailable, T12- and L3-based body composition parameters should not be considered interchangeable for longitudinal risk assessment. In parallel, baseline immunonutritional reserve—best reflected by the GNRI—was meaningfully associated with initial muscle status as well as longitudinal muscle changes and independently contributed to the stratification of toxicity and survival risk.

Although significant compositional changes were observed at both the L3 and T12 vertebral levels following chemotherapy in older patients with mCRC (Table 1), the pattern of these changes differed between the two levels (Figure 2). T12-derived SMI values were consistently lower than L3-derived SMI values, as evidenced by a negative bias across the range of measurements. Agreement differed across tissue compartments, with SATI exhibiting the highest level of concordance, while SMI and VSR showed comparatively weaker agreement. This pattern likely reflects true cross-level heterogeneity rather than measurement noise alone. L3 and T12 do not capture anatomically equivalent tissue compartments: at L3, skeletal muscle segmentation includes the psoas, quadratus lumborum, abdominal wall, and paraspinal muscles, whereas at T12 the measured muscle compartment is composed predominantly of paraspinal and trunk musculature, with abdominal wall muscles only variably represented. These anatomical differences, together with segmentation-related variability and the greater instability of ratio-based parameters, likely contribute to the negative bias of T12-SMI relative to L3-SMI and to the weaker agreement observed for SMI and VSR compared with SATI. These sources of heterogeneity should therefore be considered when interpreting both agreement metrics and the relative prognostic performance of T12- versus L3-derived measurements. Although studies in other oncologic contexts have reported moderate to strong correlations between T12- and L3-based muscle indices, these same studies caution that correlation alone does not imply interchangeability, particularly for features sensitive to regional anatomy or image segmentation error, such as ectopic fat or muscle quality parameters [13]. Huang et al. reported a significant correlation between body composition measurements obtained at T12 and L3 in patients with esophageal squamous cell carcinoma treated with chemoradiotherapy. After adjustment for age, sex, and tumor stage, low post-chemoradiotherapy T12 SMI was significantly associated with decreased overall survival and was also correlated with low post-CRT L3 SMI [10]. In a study by Arayne et al. involving patients with rectal cancer, preoperative CT-derived skeletal muscle measurements at T4 and T12 were compared with those obtained at L3. The authors reported proportional biases at the T12 level and demonstrated that thoracic vertebral levels do not behave equivalently when substituted for L3. Despite these discrepancies, the study suggested that thoracic-level measurements may still be meaningfully associated with clinical outcomes [27]. While Bland–Altman analysis and the existing literature suggest that T12 is a biologically plausible prognostic reference level after systemic therapy, they do not address the question of whether longitudinal changes at L3 or T12 are more prognostically informative, particularly in older patients with mCRC. However, the findings of the present study indicate that, although T12-derived measurements were correlated with L3-derived values, the observed systematic bias and level-specific prognostic differences do not support the use of T12-derived measurements as a substitute for the standard L3 reference level in longitudinal risk prediction.

In patients with CRC, comparisons between single-slice and multi-slice approaches suggest broadly similar cross-sectional survival associations; however, evidence remains limited with respect to longitudinal change and individual-level risk stratification [28]. One of the key findings of this study was that dynamic muscle changes appeared to be more prognostically relevant than static measures of body composition alone. ΔL3-SMI demonstrated high discriminative performance for mortality (AUC ~0.85) with an optimal cut-off around a ≥10% decline (Supplementary Table S7), and remained independently associated with mortality in multivariable analysis (Table 5). This aligns closely with prior mCRC evidence showing that >5–14% skeletal muscle loss at ~3 months identifies patients at substantially higher risk of death, even when controlling for other prognostic factors and despite weight-based measures being less informative [9,29]. Multiple recent colorectal/rectal cancer cohorts continue to support the prognostic relevance of L3-derived muscle indices and muscle radiodensity. In a rectal cancer surgical cohort, higher L3 SMI was associated with better overall survival and disease-free survival [7]. In a cohort of 226 mCRC patients, >5% skeletal muscle loss at 3 months and at 12 months after metastatic diagnosis was associated with worse overall survival, while weight loss was not associated with survival—supporting the argument that serial CT-derived muscle trajectories provide prognostic information beyond traditional metrics [9]. Additional colorectal liver metastasis data similarly indicate that early decreases in lumbar muscle metrics during chemotherapy are associated with poorer survival outcomes and may not be detectable by weight monitoring alone [8]. These observations also fit the broader CRC literature in which CT-defined sarcopenia is consistently linked to adverse survival, reinforcing the concept that muscle depletion is not merely a correlate of advanced disease but a clinically relevant host phenotype [3].

In addition to survival, early muscle loss was closely linked to treatment tolerability. Patients who developed grade ≥ 3 adverse events exhibited lower baseline albumin, PNI, and GNRI (Supplementary Table S4), and—critically—greater post-chemotherapy decline in L3-SMI (Table 2). When baseline nutritional indices and longitudinal CT changes were jointly modeled, GNRI and ΔL3-SMI remained independent predictors of severe toxicity (Table 3). In a multicenter mCRC study, Barret et al. reported that CT-defined sarcopenia was the only factor independently associated with grade 3–4 chemotherapy toxicities [30]. Longitudinal evidence also supports that muscle loss during treatment is a key determinant of tolerability: the CAIRO3 analysis showed that skeletal muscle loss during therapy was associated with a higher risk of dose-limiting toxicities and subsequent dose reductions, whereas BMI was not informative for this purpose [31]. Similarly, the AGEO prospective multicenter study emphasized that early SMI decline during systemic treatment carries prognostic relevance and is linked to higher toxicity burden [29]. Notably, a recent AI-enabled analysis in adults receiving first-line FOLFOX/FOLFIRI demonstrated that low baseline SMI was associated with a higher incidence of grade ≥ 3 neutropenia and lower 5-FU bolus relative dose intensity, reinforcing the clinical plausibility of muscle-informed toxicity risk stratification in mCRC [32].

Beyond muscle quantity, deterioration in muscle quality also demonstrated clear clinical relevance. In patients who died, IMATI increased significantly after chemotherapy at both vertebral levels, whereas survivors exhibited relatively stable IMATI values (Table 4). This observation is consistent with accumulating evidence that ectopic fat infiltration of skeletal muscle—commonly referred to as myosteatosis—is associated with adverse outcomes in CRC [33,34,35]. Mechanistically, increasing intramuscular adiposity may reflect impaired mitochondrial function, insulin resistance, inflammation-driven lipid redistribution, and reduced physical activity [36]. These alterations may progress independently of changes in visceral adiposity, as IMATI increased even in the context of declining VATI (Table 4), suggesting that muscle quality deterioration represents a distinct and clinically meaningful dimension of frailty progression during chemotherapy. Consistent with this interpretation, IMATI increased among patients who developed grade ≥ 3 toxicity (Table 2), while remaining comparatively stable in those without severe toxicity, indicating that muscle quality deterioration may accompany early muscle wasting. Beyond radiodensity-based definitions, studies using intermuscular/intramuscular fat metrics similarly report adverse prognostic associations; for example, intermuscular fat has been shown to independently predict worse long-term outcomes in rectal cancer [7]. In addition, a large meta-analysis across cancers reported that high intramuscular adipose tissue content is associated with poorer prognosis, including within CRC subgroups [37]. Taken together, these findings support a “two-hit” phenotype characterized by concurrent loss of muscle mass (SMI decline) and increasing intramuscular fat infiltration (IMATI rise), which may help explain why some older adults experience disproportionate treatment toxicity despite similar BMI and baseline clinical characteristics. IMATI may therefore be viewed as a complementary, mechanistically informative marker. Changes in SMI may reflect the loss of muscle mass (reserve depletion), whereas changes in IMATI may indicate impaired muscle quality (metabolic/functional deterioration).

To the best of our knowledge, this is also the first study to investigate the association between baseline nutritional indices and dynamic changes in CT-based body composition in older patients with mCRC. In this study, GNRI was independently associated with toxicity and mortality (Table 3 and Table 5), and it was also associated with baseline SMI and longitudinal changes in SMI at the L3 and T12 levels (Supplementary Table S2). In line with these findings, Güç et al. evaluated CT-defined sarcopenia in conjunction with multiple inflammation- and nutrition-based indices, including GNRI and PNI, in a cohort of 185 patients, and demonstrated that low GNRI and high CONUT were independent predictors of sarcopenia [38]. Similarly, Wang et al. reported that L3-derived SMI contributed meaningfully to preoperative nutritional risk stratification in colorectal cancer, identifying PNI, BMI, and L3 SMI as independent predictors of nutritional risk, while low L3 SMI was associated with poorer disease-free and overall survival [15]. In older colorectal neoplasia settings, Hisada et al. further integrated immunonutritional indices (PNI and GNRI) with CT-based muscle parameters (SMI and IMAC) in an older endoscopic cohort, demonstrating the feasibility of combined “immunonutrition plus CT muscle phenotype” models even in older populations, albeit with clinical endpoints focused primarily on postoperative complications rather than long-term survival [14]. Collectively, these studies support the concept that nutritional indices and CT-derived muscle metrics capture complementary dimensions of host vulnerability, reinforcing the biological and clinical relevance of integrating baseline nutritional status with dynamic body composition changes in older oncology.

### 4.1. Limitations

This study has several limitations that should be acknowledged. First, its retrospective and single-center design may introduce selection bias and limit the generalizability of the findings. Additionally, given the retrospective observational design, the observed associations should not be interpreted as indicating causality. The relatively modest sample size, although comparable to prior longitudinal CT-based body composition studies in metastatic colorectal cancer, may have reduced statistical power for subgroup analyses and increased the risk of overfitting despite the use of penalized regression techniques. Second, the study population consisted exclusively of older patients with metastatic colorectal cancer receiving systemic chemotherapy. Therefore, the results may not be directly applicable to younger patients, patients with non-metastatic disease, or those treated with curative intent. Although multivariable regression models were adjusted for several clinical covariates, the possibility of residual confounding from unmeasured or incompletely captured factors cannot be fully excluded. Third, CT-derived body composition analysis relied on single-slice measurements at predefined vertebral levels. While this methodology is well validated and widely used, it may not fully capture whole-body muscle and adipose tissue distribution. In addition, agreement between T12- and L3-derived measurements was not uniform across all compartments, indicating potential measurement variability related to anatomical level, tissue segmentation, and calibration error. Such variability should be considered when interpreting the magnitude of associations observed for thoracic versus lumbar body composition parameters. Fourth, CT-based metrics primarily reflect muscle quantity and radiologic fat infiltration and do not incorporate functional aspects of sarcopenia, such as muscle strength, physical performance, or patient-reported outcomes. Therefore, the multidimensional clinical phenotype of frailty and functional decline may have been only partially captured. Importantly, nutritional indices (PNI and GNRI) were assessed only at baseline, and their temporal changes during chemotherapy were not evaluated. Dynamic alterations in nutritional and inflammatory status over the course of treatment may provide additional prognostic information and could potentially modify the relationship between baseline nutritional reserve, longitudinal muscle loss, and clinical outcomes. The absence of serial measurements of nutritional indices therefore limits the ability to fully characterize the bidirectional interplay between evolving nutritional status and CT-derived body composition changes. Finally, data on dietary intake, physical activity, systemic inflammatory burden over time, and structured nutritional or exercise interventions were limited. These unmeasured factors may have influenced longitudinal body composition trajectories and clinical outcomes. Accordingly, the present findings should be interpreted as supportive of risk stratification rather than evidence of preventive or therapeutic benefit. Prospective studies incorporating serial nutritional assessments, functional measures, and interventional strategies are warranted to validate and extend these findings.

### 4.2. Future Directions

Future research should aim to prospectively validate the present findings in larger, multicenter cohorts of older patients with metastatic colorectal cancer. In particular, longitudinal studies incorporating repeated assessments of nutritional indices, inflammatory biomarkers, muscle strength, and physical performance may help clarify the dynamic interaction between immunonutritional reserve, treatment-related muscle loss, and clinical outcomes. Another important area of investigation will be the development of standardized approaches for thoracic vertebral body composition assessment. Although the present study demonstrates that T12-derived measurements correlate with L3-derived metrics, systematic bias limits their direct interchangeability. Future studies may explore calibration models or segmentation approaches that could enable reliable use of thoracic CT levels in clinical settings where L3 images are not consistently available. Finally, interventional studies are warranted to determine whether early identification of patients with significant skeletal muscle loss or increasing intramuscular adiposity can support targeted supportive strategies. These may include individualized nutritional interventions, exercise-based rehabilitation programs, or treatment-adaptation strategies aimed at improving chemotherapy tolerance and long-term survival in vulnerable older populations.

## 5. Conclusions

In older patients with mCRC, early post-chemotherapy skeletal muscle loss—particularly at the L3 level—emerged as the most powerful imaging-based predictor of severe toxicity and mortality, exceeding the prognostic value of baseline body composition alone. GNRI provided complementary prognostic information, reflecting baseline immunonutritional reserve and its relationship with subsequent muscle deterioration. Although T12-derived measurements showed correlations with L3-derived values, the observed systematic bias indicates that thoracic measurements cannot fully replace the standard L3 reference level for longitudinal risk assessment. These findings suggest that routine CT-based muscle monitoring combined with nutritional indices may have value for risk stratification, but causal or therapeutic implications cannot be inferred from this retrospective observational study.

## Figures and Tables

**Table 1:** Changes in CT-based body composition and correlation with baseline nutritional indices.

Variables	Chemotherapy		*p*-Value	PNI		GNRI	
	Before n = 87	After n = 87		r	*p*-Value	r	*p*-Value
L3							
SMI	46.7 ± 8.8	42.8 ± 9.8	<0.001 *	0.302	0.033 *	0.502	<0.001 *
SATI	57.2 (35.4–75.2)	51.9 (35.8–91.8)	0.856	0.110	0.310	0.038	0.727
VATI	55.0 (36.0–84.2)	47.0 (27.7–69.2)	<0.001 *	0.047	0.664	0.064	0.558
VSR	1.0 (0.7–1.4)	0.9 (0.5–1.3)	<0.001 *	0.038	0.728	0.059	0.588
IMATI	5.3 (4.0–8.8)	6.1 (4.5–9.6)	0.034 *	−0.294	0.139	−0.344	0.016 *
T12							
SMI	34.6 ± 8.2	31.6 ± 8.1	<0.001 *	0.043	0.692	0.317	0.025 *
SATI	35.2 (20.4–53.5)	32.3 (22.0–57.4)	0.535	0.117	0.282	0.065	0.552
VATI	36.7 (19.8–60.1)	31.8 (15.0–48.1)	0.001 *	0.049	0.655	0.161	0.137
VSR	1.0 (0.6–1.6)	0.9 (0.4–1.4)	<0.001 *	0.142	0.191	0.070	0.522
IMATI	3.4 (2.0–5.5)	4.4 (2.5–5.8)	0.020 *	−0.287	0.201	−0.309	0.035 *

Data are mean ± standard deviation or median (IQR). * *p*-value < 0.05 indicates statistical significance. Abbreviations: GNRI, geriatric nutritional risk index; IMATI, intramuscular adipose tissue index; L3, the third lumbar vertebra; PNI, prognostic nutritional index; SATI, subcutaneous adipose tissue index; SMI, skeletal muscle index; VATI, visceral adipose tissue index; VSR, visceral-to-subcutaneous fat ratio; T12, the twelfth thoracic vertebra.

**Table 2:** CT-based body composition changes at L3 and T12 vertebral levels according to grade ≥ 3 adverse events.

Variables	Grade ≥ 3 Adverse Event (−)		*p*-Value	Grade ≥ 3 Adverse Event (+)		*p*-Value	Δ *p*-Value
	Before CTx	After CTx		Before CTx	After CTx		
L3							
SMI	46.5 ± 8.5	43.6 ± 8.7	<0.001 *	42.0 ± 7.2	36.4 ± 8.3	<0.001 *	<0.001 *
SATI	51.2 (34.5–74.9)	49.6 (36.1–90.3)	0.669	60.3 (51.0–86.8)	55.8 (34.7–107.6)	0.670	0.895
VATI	54.9 (36.2–77.2)	46.8 (28.6–69.2)	0.007 *	61.2 (36–93.5)	48.5 (24.3–80.4)	0.001 *	<0.001 *
VSR	1.0 (0.7–1.4)	0.9 (0.5–1.3)	0.038 *	0.9 (0.7–1.5)	0.7 (0.5–1.1)	0.008 *	0.032 *
IMATI	5.4 (4.1–8.7)	5.6 (3.9–8.5)	0.563	5.3 (3.2–9.2)	6.1 (3.9–11.3)	0.047 *	0.021 *
T12							
SMI	33.7 ± 7.8	31.3 ± 7.1	<0.001 *	37.2 ± 9.1	32.3 ± 10.6	0.002 *	0.024 *
SATI	30.5 (20.3–48.5)	31 (20.6–56.3)	0.593	38.4 (20.8–60.2)	40.0 (25.2–63.0)	0.808	0.526
VATI	34.6 (19.8–60.1)	31.3 (14.5–48.9)	0.024 *	43 (18.8–64)	37.9 (17.0–47.0)	0.005 *	0.128
VSR	1.1 (0.6–1.7)	0.9 (0.4–1.5)	0.004 *	1.0 (0.7–1.6)	0.7 (0.3–1.5)	0.013 *	0.808
IMATI	3.4 (2.3–5.5)	4.0 (2.5–5.8)	0.116	3.3 (1.9–6.9)	4.5 (2.4–7.0)	0.046 *	0.038 *

Data are mean ± standard deviation or median (IQR). * *p*-value < 0.05 indicates statistical significance. Δ *p*-value indicates the comparison of change between groups. Abbreviations: CTx, chemotherapy; L3, the third lumbar vertebra; SATI, subcutaneous adipose tissue index; SMI, skeletal muscle index; VATI, visceral adipose tissue index; VSR, visceral-to-subcutaneous fat ratio; IMATI, intramuscular adipose tissue index; T12, the twelfth thoracic vertebra.

**Table 3:** Independent predictors of grade ≥ 3 adverse events.

Variables	Univariable Regression		VIF	Multivariable Regression	
	OR (95% CI)	*p*-Value		OR (95% CI)	*p*-Value
ONS	2.75 (1.05–7.47)	0.048 *	1.09	–	–
Albumin	0.15 (0.05–0.25)	0.008 *	2.61	–	–
PNI	0.88 (0.80–0.96)	0.006 *	1.70	–	–
GNRI	0.85 (0.77–0.94)	0.001 *	2.32	0.86 (0.78–0.94)	0.002 *
L3 SMI	0.93 (0.88–0.99)	0.042 *	1.25	–	–
ΔL3 SMI	0.85 (0.79–0.92)	<0.001 *	1.72	0.87 (0.81–0.94)	0.001 *
ΔL3 VSR	0.95 (0.91–0.99)	0.036 *	1.15	–	–
ΔL3 IMATI	1.04 (1.02–1.07)	0.025 *	1.37	–	–
ΔT12 SMI	0.94 (0.90–0.98)	0.026 *	1.45	–	–
ΔT12 IMATI	1.06 (1.01–1.12)	0.046 *	1.46	–	–
				Nagelkerke R^2^ = 0.45	

Age, gender, BMI, comorbidity burden, site of metastasis, surgical operation, lymphovascular invasion, perineural invasion, chemotherapy regime and number of cycles were adjusted in multivariable analyses. * *p*-value < 0.05 indicates statistical significance. Abbreviations: CI, confidence interval; GNRI, geriatric nutritional risk index; ONS, oral nutritional support; SMI, skeletal muscle index; IMATI, intramuscular adipose tissue index; VIF, variance inflation factor; VSR, visceral-to-subcutaneous fat ratio; PNI, prognostic nutritional index; Δ, post-chemotherapy change.

**Table 4:** CT-based body composition changes at L3 and T12 vertebral levels according to survival.

Variables	Alive		*p*-Value	Deceased		*p*-Value	Δ*p*-Value
	Before CTx	After CTx		Before CTx	After CTx		
L3							
SMI	52.3 ± 11.0	51.7 ± 13.0	0.691	45.6 ± 7.9	40.9 ± 8.0	<0.001 *	<0.001 *
SATI	59.5 (25.8–86.8)	62.6 (41.0–94.5)	0.191	53.7 (35.5–74.3)	49.5 (34.8–85.8)	0.734	0.262
VATI	73 (25.5–108.1)	69.2 (27.7–103.6)	0.021 *	54.9 (36.2–77.2)	45.2 (28.1–62.9)	<0.001 *	0.297
VSR	1.0 (0.7–1.5)	0.9 (0.5–1.3)	0.022 *	1.0 (0.7–1.4)	0.9 (0.6–1.2)	<0.001 *	0.975
IMATI	4.5 (3.2–8.1)	5.0 (2.8–10.2)	0.139	5.0 (4.0–8.4)	6.1 (4.5–10.8)	0.024 *	0.001 *
T12							
SMI	38.2 ± 11.3	36.5 ± 10.5	0.037 *	33.9 ± 7.4	29.6 ± 7.2	<0.001 *	0.011 *
SATI	40.5 (15.8–75.7)	46.3 (24.2–73.1)	0.433	31.6 (20.7–45)	31.0 (21.7–56.9)	0.753	0.620
VATI	43.1 (19.9–78.7)	38.7 (14.5–61.4)	0.009 *	35.6 (18.8–55.6)	31.8 (16.2–46.7)	0.011 *	0.102
VSR	1.2 (1.0–1.9)	0.9 (0.3–1.3)	0.010 *	1.0 (0.6–1.7)	0.8 (0.4–1.5)	0.004 *	0.113
IMATI	3.8 (1.2–10.7)	4.2 (2.2–8.4)	0.306	4.0 (2.3–6.5)	5.2 (2.7–8.6)	0.036 *	0.007 *

Data are mean ± standard deviation or median (IQR). * *p*-value < 0.05 indicates statistical significance. Δ *p*-value indicates the comparison of change between groups. Abbreviations: L3, the third lumbar vertebra; SATI, subcutaneous adipose tissue index; SMI, skeletal muscle index; VATI, visceral adipose tissue index; VSR, visceral-to-subcutaneous fat ratio; IMATI, intramuscular adipose tissue index; T12, the twelfth thoracic vertebra.

**Table 5:** Independent predictors of mortality.

Variables	Univariable Regression		VIF	Multivariable Regression	
	HR (95% CI)	*p*-Value		HR (95% CI)	*p*-Value
ECOG 2	2.62 (1.04–6.64)	0.042 *	1.24	–	–
Surgical operation	0.59 (0.36–0.97)	0.036 *	1.26	–	–
Number of cycles	0.94 (0.90–0.99)	0.019 *	2.73	–	–
PFS	0.95 (0.90–0.99)	0.008 *	2.70	0.94 (0.90–0.98)	0.036 *
Albumin	0.39 (0.21–0.73)	0.003 *	2.72	–	–
CRP	1.03 (1.01–1.05)	0.002 *	1.18	1.08 (1.02–1.14)	0.012 *
PNI	0.97 (0.94–0.99)	0.013 *	1.74	–	–
GNRI	0.96 (0.93–0.99)	<0.001 *	2.35	0.96 (0.93–0.99)	0.024 *
Baseline L3 SMI	0.96 (0.94–0.99)	<0.001 *	1.27	–	–
ΔL3 SMI	0.97 (0.95–0.99)	<0.001 *	1.26	0.97 (0.95–0.99)	0.007 *
ΔL3 IMATI	1.03 (1.01–1.08)	0.018 *	1.52	–	–
ΔT12 SMI	0.98 (0.97–0.99)	0.027 *	1.53	–	–
ΔT12 IMATI	1.04 (1.01–1.09)	0.024 *	1.67	–	–
				−2 Log Likelihood = 307.6	

Age, gender, body mass index, comorbidity burden, site of metastasis, surgical operation, lymphovascular invasion, perineural invasion, and chemotherapy regime were adjusted in multivariable analyses. * *p*-value < 0.05 indicates statistical significance. Abbreviations: CRP, C-reactive protein; ECOG, Eastern Cooperative Oncology Group; GNRI, geriatric nutritional risk index; IMATI, intramuscular adipose tissue index; L3, the third lumbar vertebra; PFS, progression-free survival; PNI, prognostic nutritional index; SMI, skeletal muscle index; VIF, variance inflation factor; Δ, post-chemotherapy change.

## Data Availability

The data that support the findings of this study are available on request from the corresponding author due to privacy and ethical reasons.

## Supplementary Materials

The following supporting information can be downloaded at: https://www.mdpi.com/article/10.3390/nu18071090/s1, Table S1: Demographic and clinical characteristics of older patients with mCRC; Table S2: Association of baseline PNI and GNRI with CT-based body composition parameters at L3 and T12 vertebral levels; Table S3: Demographic and clinical characteristics by Grade ≥ 3 adverse events; Table S4: Laboratory and CT-based body composition findings by Grade ≥ 3 adverse event; Table S5: Demographic and clinical parameters associated with mortality; Table S6: Laboratory and CT-based body composition parameters associated with mortality; Table S7: Diagnostic performance of nutritional and CT-based body composition parameters in predicting mortality.
