# Supplementary material for: Post-Chemotherapy Changes and Agreement of CT-Derived Body Composition at L3 and T12 in Older Patients with Metastatic Colorectal Cancer: Associations with Nutritional Indices and Outcomes
Source: Nutrients. 2026 Mar 28;18(7):1090. doi: 10.3390/nu18071090 (PMC13074540; doi:10.3390/nu18071090)
Supplement: Supplementary file 1 [file nutrients-18-01090-s001.zip › Supplement Tables-revised.pdf]

Supplement Table S1. Demographic and clinical characteristics of older patients with mCRC.

Variables	All population n = 87
Age, years	69.6 ± 4.9
Male gender, n (%)	59 (67.8)
Weight, kg	72.3 ± 14.8
Length, m	1.7 ± 0.1
BMI, kg/m^2	26.1 ± 4.6
Smoking, n (%)	29 (33.3)
Comorbidity, n (%)	42 (48.3)
Hypertension	26 (29.9)
Diabetes mellitus	15 (17.2)
CHF	2 (2.3)
CAD	7 (8.0)
COPD	1 (1.1)
CCI	9 (8 – 10)
ECOG 2	5 (5.7)
TNM stage IV, n (%)	87 (100)
T stage, n (%)	
1	19 (21.8)
2	68 (78.2)
N stage, n (%)	
0	8 (9.2)
1	16 (18.4)
2	63 (72.4)
M stage, n (%)	87 (100)
Site of metastasis, n (%)	
Lymph node	78 (89.7)
Liver	51 (58.6)
Lung	20 (23.0)
Peritoneum	12 (13.8)
ONS support, n (%)	24 (27.6)
Surgical operation, n (%)	59 (67.8)
Lymphovascular invasion, n (%)	51 (58.6)
Perineural invasion, n (%)	44 (50.6)
Ostomy, n (%)	27 (31.0)
CTx regime, n (%)	
Oxaliplatin	76 (87.4)
Irinotecan	8 (9.2)
Capecitabine	3 (3.4)
Number of cycles	8.0 (6.0 – 12.0)
Radiotherapy, n (%)	5 (5.7)
PFS, months	7.6 (5.3 – 10.3)
Mortality, n (%)	72 (82.8)
Follow-time, months	25.2 (16.3 – 41.5)

Data are mean ± standard deviation or median (IQR), or number (%). *p-value < 0.05 indicates statistical significance. Abbreviations: CRC, colorectal cancer; BMI, body mass index; CHF, congestive heart failure; CAD, coronary artery disease; COPD, chronic obstructive pulmonary disease; CCI, Charlson Comorbidity Index; ECOG, Eastern Cooperative Oncology Group; TNM, tumor–node–metastasis; ONS, oral nutritional support; IVI, intravenous iron; CTX, chemotherapy; PFS, progression-free survival.

Supplement Table S2. Association of baseline PNI and GNRI with CT-based body composition parameters at L3 and T12 vertebral levels.

Outcome	Crude analysis			Adjusted analysis		
	**β ± SE**	**95% CI**	***p*-value**	**β ± SE**	**95% CI**	***p*-value**
Before chemotherapy						
L3						
SMI	0.60 ± 0.16	0.28 to 0.93	<0.001*	0.64 ± 0.17	0.29 to 0.98	<0.001*
SATI	0.57 ± 0.75	-0.93 to 2.06	0.454	0.47 ± 0.79	-1.09 to 2.04	0.547
VATI	0.46 ± 0.67	-0.87 to 1.79	0.495	0.43 ± 0.71	-0.98 to 1.85	0.546
VSR	0.01 ± 0.01	-0.03 to 0.01	0.356	0.01 ± 0.01	-0.03 to 0.01	0.363
IMATI	-0.22 ± 0.07	-0.36 to -0.08	0.016*	0.24 ± 0.11	-0.46 to -0.02	0.035*
T12						
SMI	0.32 ± 0.12	0.08 to 0.56	0.014*	0.35 ± 0.15	0.05 to 0.50	0.035*
SATI	1.09 ± 0.67	-0.23 to 2.42	0.104	1.14 ± 0.69	-0.24 to 2.53	0.104
VATI	0.74 ± 0.54	-0.34 to 1.82	0.178	0.75 ± 0.58	-0.42 to 1.91	0.205
VSR	-0.01 ± 0.02	-0.05 to 0.04	0.834	0.01 ± 0.02	-0.05 to 0.04	0.800
IMATI	0.18 ± 0.07	-0.35 to -0.04	0.020*	0.21 ± 0.09	-0.39 to -0.03	0.039*
Change after chemotherapy						
ΔL3						
SMI	0.54 ± 0.18	0.18 to 0.90	0.004*	0.51 ± 0.18	0.14 to 0.87	0.007*
SATI	1.28 ± 1.38	-1.46 to 4.02	0.355	1.42 ± 1.37	-1.32 to 4.15	0.306
VATI	1.34 ± 0.90	-0.44 to 3.12	0.139	1.62 ± 0.94	-0.25 to 3.49	0.089
VSR	0.81 ± 0.81	-0.81 to 2.42	0.324	1.23 ± 0.84	-0.45 to 2.90	0.148
IMATI	-0.37 ± 0.16	-0.69 to -0.05	0.013*	0.42 ± 0.19	-0.80 to -0.04	0.045*
ΔT12						
SMI	0.37 ± 0.14	0.09 to 0.65	0.035*	0.42 ± 0.18	0.06 to 0.78	0.047*
SATI	-1.00 ± 1.01	-3.00 to 1.00	0.321	-1.16 ± 1.02	-3.19 to 0.87	0.258
VATI	-0.97 ± 1.39	-3.73 to 1.79	0.487	-1.06 ± 1.42	-3.90 to 1.77	0.457
VSR	-0.64 ± 1.17	-2.97 to 1.69	0.586	-0.47 ± 1.19	-2.85 to 1.90	0.694
IMATI	-0.28 ± 0.11	-0.40 to -0.06	0.026*	-0.33 ± 0.15	-0.63 to -0.03	0.042*

Age, gender, BMI, comorbidity burden, ECOG, site of metastasis, ONS, support, surgical operation, lymphovascular invasion, perineural invasion, chemotherapy regime, number of cycles, and other laboratory parameters were adjusted in adjusted analysis. *p-value < 0.05 indicates statistical significance. Abbreviations: SMI, skeletal muscle index; SATI, subcu-taneous adipose tissue index; VATI, visceral adipose tissue index; VSR, visceral-to-subcutaneous fat ratio; IMATI, intramuscular adipose tissue index; PNI, prognostic nutritional index; GNRI, geriatric nutritional risk index.

Supplement Table S3. Demographic and clinical characteristics by Grade ≥3 adverse events.

**Variables**	**Grade ≥3 adverse events**		***p*-value**
	**No** **n=64**	**Yes** **n=23**	
Age, years	69.9 ± 4.4	70.0 ± 4.6	0.379
Male gender, n (%)	44 (68.8)	15 (65.2)	0.548
Weight, kg	73.1 ± 14.7	70.3 ± 15.4	0.451
Length, m	1.7 ± 0.1	1.7 ± 0.1	0.866
BMI, kg/m^2	26.3 ± 4.4	25.7 ± 5.2	0.591
Smoking, n (%)	24 (37.5)	5 (21.7)	0.169
Comorbidity, n (%)	34 (53.1)	8 (34.8)	0.131
Hypertension	21 (32.8)	5 (21.7)	0.320
Diabetes mellitus	13 (20.3)	2 (8.7)	0.206
CHF	1 (1.6)	1 (4.3)	0.445
CAD	5 (7.8)	2 (8.7)	0.894
COPD	1 (1.6)	0	0.547
CCI	9 (8 – 10)	9 (8 – 10)	0.307
ECOG 2	2 (3.1)	3 (13.0)	0.080
TNM stage IV, n (%)	64 (100)	23 (100)	0.999
T stage, n (%)			
1	17 (26.6)	2 (8.7)	0.199
2	47 (73.4)	21 (91.3)	
N stage, n (%)			
0	7 (10.9)	1 (4.3)	
1	12 (18.8)	4 (17.4)	0.304
2	45 (70.3)	18 (78.3)	
M stage, n (%)	64 (100)	23 (100)	0.999
Site of metastasis, n (%)			
Lymph node	56 (87.5)	22 (95.7)	0.271
Liver	37 (57.8)	14 (60.9)	0.798
Lung	14 (21.9)	6 (26.1)	0.681
Peritoneum	10 (15.6)	2 (8.7)	0.408
ONS support, n (%)	14 (21.9)	10 (43.5)	0.047*
Surgical operation, n (%)	47 (73.4)	12 (52.2)	0.061
Lymphovascular invasion, n (%)	40 (62.5)	11 (47.8)	0.220
Perineural invasion, n (%)	34 (53.1)	10 (43.5)	0.427
Ostomy, n (%)	19 (29.7)	8 (34.8)	0.651
CTx regime, n (%)			
Oxaliplatin	57 (89.1)	19 (82.6)	
Irinotecan	5 (7.8)	3 (13.0)	0.720
Capecitabine	2 (3.1)	1 (4.3)	
Number of cycles	8.0 (6.0 – 12.0)	9.0 (6.0 – 12.0)	0.812
Radiotherapy, n (%)	2 (3.1)	3 (13.0)	0.080
PFS, months	7.2 (5.3 – 10.3)	7.9 (5.6 – 10.9)	0.456
Mortality, n (%)	52 (81.2)	20 (87.0)	0.534
Follow-time, months	26.8 (16.2 – 41.7)	22.6 (16.6 – 39.3)	0.610

Data are mean ± standard deviation or median (IQR), or number (%). *p-value < 0.05 indicates statistical significance. Abbreviations: CRC, colorectal cancer; BMI, body mass index; CHF, congestive heart failure; CAD, coronary artery disease; COPD, chronic obstructive pulmonary disease; CCI, Charlson Comorbidity Index; ECOG, Eastern Cooperative Oncology Group; TNM, tumor–node–metastasis; ONS, oral nutritional support; IVI, intravenous iron; CTX, chemotherapy; PFS, progression-free survival.

Supplement Table S4. Laboratory and CT-based body composition findings by Grade ≥3 adverse event.

Variables	Grade ≥3 adverse event		*p*-value
	**No** n=64	**Yes** n=23	
Laboratory findings			
Hemoglobin, g/dL	11.8 ± 2.1	11.8 ± 1.4	0.922
Neutrophils, $\times 10^9$/L	4.6 ± 1.8	4.7 ± 2.0	0.708
Platelets, $\times 10^9$/L	276.5 (206.0 – 390.0)	288.0 (210.5 – 376.0)	0.740
Lymphocyte, $\times 10^9$/L	1.6 (1.1 – 2.0)	1.6 (1.1 – 2.5)	0.992
Creatinine, mg/L	0.9 (0.7 – 1.0)	0.8 (0.7 – 1.0)	0.418
AST, U/L	17.5 (13.0 – 20.0)	15.0 (12.5 – 18.5)	0.269
ALT, U/L	15.5 ± 6.3	14.0 ± 6.2	0.328
Albumin, g/dL	3.7 ± 0.4	3.4 ± 0.5	0.005*
CRP, mg/L	10.0 (2.0 – 31.0)	13.4 (5.0 – 32.0)	0.190
PNI	45.6 ± 8.0	40.4 ± 5.7	0.006*
GNRI	94.7 ± 6.2	89.2 ± 5.1	<0.001*
CT findings			
Before chemotherapy			
L3			
SMI	46.5 ± 8.5	42.0 ± 7.2	0.033*
SATI	51.2 (34.5 – 74.9)	60.3 (51.0 – 86.8)	0.126
VATI	54.9 (36.2 – 77.2)	61.2 (36 – 93.5)	0.296
VSR	1.0 (0.7 – 1.4)	0.9 (0.7 – 1.5)	0.683
IMATI	5.4 (4.1 – 8.7)	5.3 (3.2 – 9.2)	0.862
T12			
SMI	33.7 ± 7.8	37.2 ± 9.1	0.083
SATI	30.5 (20.3 – 48.5)	38.4 (20.8 – 60.2)	0.161
VATI	34.6 (19.8 – 60.1)	43 (18.8 – 64)	0.266
VSR	1.1 (0.6 – 1.7)	1.0 (0.7 – 1.6)	0.996
IMATI	3.4 (2.3 – 5.5)	3.3 (1.9 – 6.9)	0.908
After chemotherapy			
L3			
SMI	43.6 ± 8.7	36.4 ± 8.3	<0.001*
SATI	49.6 (36.1 – 90.3)	55.8 (34.7 – 107.6)	0.551
VATI	46.8 (28.6 – 69.2)	48.5 (24.3 – 80.4)	0.746
VSR	0.9 (0.5 – 1.3)	0.7 (0.5 – 1.1)	0.681
IMATI	5.6 (3.9 – 8.5)	6.1 (3.9 – 11.3)	0.324
T12			
SMI	31.3 ± 7.1	32.3 ± 10.6	0.703
SATI	31 (20.6 – 56.3)	40.0 (25.2 – 63.0)	0.156
VATI	31.3 (14.5 – 48.9)	37.9 (17.0 – 47.0)	0.886
VSR	0.9 (0.4 – 1.5)	0.7 (0.3 – 1.5)	0.388
IMATI	4.0 (2.5 – 5.8)	4.5 (2.4 – 7.0)	0.747

Data are mean ± standard deviation or median (IQR), or number (%). *p-value < 0.05 indicates statistical significance. Abbreviations: AST, aspartate aminotransferase; ALT, alanine aminotransferase; CRP, C-reactive protein; PNI, prognostic nutritional index; GNRI, geriatric nutritional risk index; SMI, skeletal muscle index; SATI, subcutaneous adipose tissue index; VATI, visceral adipose tissue index; VSR, visceral-to-subcutaneous fat ratio; IMATI, intramuscular adipose tissue index.

Supplement Table S5. Demographic and clinical parameters associated with mortality.

Variables	Alive n=15	Deceased n=72	Univariable regression	
			**HR (95% CI)**	***p*-value**
Age, years	69.1 ± 4.7	70.3 ± 4.4	1.04 (1.00 – 1.09)	0.080
Male gender, n (%)	9 (60.0)	50 (69.4)	0.87 (0.52 – 1.44)	0.586
Weight, kg	72.3 ± 10.7	72.4 ± 15.6	0.99 (0.98 – 1.01)	0.444
Length, m	1.7 ± 0.1	1.7 ± 0.1	0.79 (0.07 – 9.05)	0.847
BMI, kg/m^2	25.8 ± 4.5	26.2 ± 4.7	0.99 (0.93 – 1.04)	0.668
Smoking, n (%)	4 (26.7)	25 (34.7)	0.73 (0.45 – 1.19)	0.210
Comorbidity, n (%)	8 (53.3)	34 (47.2)	0.80 (0.50 – 1.28)	0.353
Hypertension	5 (33.3)	21 (29.2)	0.90 (0.54 – 1.50)	0.681
Diabetes mellitus	3 (20.0)	12 (16.7)	0.94 (0.50 – 1.75)	0.837
CHF	1 (6.7)	1 (1.4)	1.46 (0.20 – 10.65)	0.710
CAD	1 (6.7)	6 (8.3)	1.59 (0.68 – 3.71)	0.286
COPD	0	1 (1.4)	0.36 (0.05 – 2.69)	0.320
CCI	8.5 ± 1.0	8.8 ± 0.9	1.23 (0.94 – 1.60)	0.131
ECOG 2	0 (0.0)	5 (6.9)	2.62 (1.04 – 6.64)	0.042*
T stage, n (%)				
1	4 (26.7)	15 (20.8)	ref	
2	11 (73.3)	57 (79.2)	1.47 (0.87 – 2.49)	0.149
N stage, n (%)				
0	3 (20.0)	5 (6.9)		
1	3 (20.0)	13 (18.1)		
2	9 (60.0)	54 (75.0)	1.35 (0.96 – 1.90)	0.089
Metastasis, n (%)				
Lymphnode	11 (73.3)	67 (93.1)	1.68 (0.67 – 4.18)	0.266
Liver	6 (40.0)	45 (62.5)	1.43 (0.89 – 2.32)	0.141
Lung	2 (13.3)	18 (25.0)	1.10 (0.64 – 1.89)	0.723
Peritoneum	6 (40.0)	6 (8.3)	0.79 (0.34 – 1.83)	0.581
ONS support, n (%)	1 (6.7)	23 (31.9)	1.12 (0.68 – 1.84)	0.666
Surgical operation, n (%)	12 (80.0)	47 (65.3)	0.59 (0.36 – 0.97)	0.036*
Lymphovascular invasion, n (%)	11 (73.3)	40 (55.6)	1.14 (0.68 – 1.91)	0.618
Perineural invasion, n (%)	10 (66.7)	34 (47.2)	0.76 (0.48 – 1.22)	0.259
Ostomy, n (%)	6 (40.0)	21 (29.2)	0.61 (0.38 – 1.07)	0.138
Chemotherapy regime, n (%)				
Oxaliplatin	13 (86.7)	63 (87.5)		
Irinotecan	2 (13.3)	6 (8.3)	1.07 (0.63 – 1.80)	0.805
Capecitabine	0	3 (4.2)	1.58 (0.49 – 5.08)	0.438
Number of cycles	10.0 (6.5 – 12.0)	8.0 (6.0 – 12.0)	0.94 (0.90 – 0.99)	0.019*
Radiotherapy, n (%)	0 (0.0)	5 (6.9)	0.86 (0.34 – 2.14)	0.739
Grade ≥3 adverse events, n (%)	3 (20.0)	20 (27.8)	1.26 (0.75 – 2.11)	0.388
Neuropathy	2 (13.3)	5 (6.9)	0.85 (0.34 – 2.13)	0.735
Neutropenia	2 (13.3)	8 (11.1)	0.91 (0.43 – 1.90)	0.799
Thrombopenia	1 (6.7)	6 (8.3)	1.65 (0.71 – 3.82)	0.244
PFS, months	7.7 (6.3 – 11.9)	7.5 (5.3 – 10.2)	0.95 (0.90 – 0.99)	0.008*
Follow-time, months	42.1 (24.9 – 73.8)	22.7 (16.2 – 39.2)	–	–

Data are mean ± standard deviation or median (IQR), or number (%). *p-value < 0.05 indicates statistical significance. Abbreviations: CRC, colorectal cancer; BMI, body mass index; CHF, congestive heart failure; CAD, coronary artery disease; COPD, chronic obstructive pulmonary disease; CCI, Charlson Comorbidity Index; ECOG, Eastern Cooperative Oncology Group; HR, hazard ratio; CI, confidence interval; ONS, oral nutritional support; IVI, intravenous iron; CTX, chemotherapy; PFS, progression-free survival.

Supplement Table S6. Laboratory and CT-based body composition parameters associated with mortality.

Variables	Alive	Deceased	Univariable regression	
	n=15	n=72	HR (95% CI)	*p*-value
Laboratuar findings				
Hemoglobin, g/dL	11.3 ± 3.2	11.9 ± 1.6	0.99 (0.90 – 1.10)	0.901
Neutrophils, ×10^9/L	4.5 (2.8 – 5.5)	4.6 (3.5 – 5.7)	0.99 (0.87 – 1.12)	0.838
Platelets, ×10^9/L	259.0 (219.0 – 390.0)	279.0 (203.0 – 379.0)	1.00 (0.99 – 1.01)	0.346
Lymphocyte, ×10^9/L	1.7 (1.2 – 2.0)	1.6 (1.1 – 2.2)	0.93 (0.72 – 1.19)	0.553
Creatinine, mg/L	0.9 (0.8 – 0.9)	0.8 (0.7 – 1.0)	0.71 (0.28 – 1.81)	0.467
AST, U/L	18.0 (13.5 – 19.5)	16.5 (13.0 – 19.2)	0.99 (0.94 – 1.04)	0.631
ALT, U/L	15.7 ± 7.2	15.0 ± 6.1	0.99 (0.95 – 1.03)	0.530
Albumin, g/dL	4.1 ± 0.3	3.5 ± 0.4	0.39 (0.21 – 0.73)	0.003*
CRP, mg/L	8.0 (2.6 – 16.5)	12.5 (4.0 – 33.3)	1.03 (1.01 – 1.05)	0.002*
PNI	46.8 ± 6.9	43.7 ± 7.8	0.97 (0.94 – 0.99)	0.013*
GNRI	97.8 ± 8.2	92.3 ± 5.6	0.96 (0.93 – 0.99)	<0.001*
CT Findings				
Before chemotherapy				
L3				
SMI	52.3 ± 11.0	45.6 ± 7.9	0.96 (0.94 – 0.99)	<0.001*
SATI	59.5 (25.8-86.8)	53.7 (35.5-74.3)	1.00 (0.99 – 1.02)	0.661
VATI	73 (25.5-108.1)	54.9 (36.2-77.2)	1.00 (0.99 – 1.01)	0.449
VSR	1.0 (0.7-1.5)	1.0 (0.7-1.4)	0.92 (0.61 – 1.38)	0.682
IMATI	4.5 (3.2-8.1)	5.0 (4.0-8.4)	1.00 (0.94 – 1.05)	0.798
T12				
SMI	38.2 ± 11.3	33.9 ± 7.4	0.99 (0.96 – 1.02)	0.493
SATI	40.5 (15.8-75.7)	31.6 (20.7-45)	1.00 (0.99 – 1.01)	0.707
VATI	43.1 (19.9-78.7)	35.6 (18.8-55.6)	1.00 (0.99 – 1.01)	0.549
VSR	1.2 (1.0-1.9)	1.0 (0.6-1.7)	0.93 (0.74 – 1.18)	0.566
IMATI	3.8 (1.2-10.7)	4.0 (2.3-7.0)	1.00 (0.93 – 1.07)	0.601
After chemotherapy				
L3				
SMI	51.7 ± 13.0	40.9 ± 8.0	0.97 (0.94 – 0.99)	<0.001*
SATI	62.6 (41.0-94.5)	49.5 (34.8-85.8)	1.00 (0.99 – 1.02)	0.740
VATI	69.2 (27.7-103.6)	45.2 (28.1-62.9)	0.99 (0.98 – 1.03)	0.168
VSR	0.9 (0.5-1.3)	0.9 (0.6-1.2)	0.76 (0.48 – 1.21)	0.245
IMATI	5.0 (2.8-10.2)	6.1 (4.5-10.8)	0.99 (0.94 – 1.05)	0.794
T12				
SMI	36.5 ± 10.5	29.6 ± 7.2	0.97 (0.95 – 1.02)	0.073
SATI	46.3 (24.2-73.1)	31.0 (21.7-56.9)	0.99 (0.96 – 1.02)	0.395
VATI	38.7 (14.5-61.4)	31.8 (16.2-46.7)	1.00 (0.99 – 1.01)	0.757
VSR	0.9 (0.3-1.3)	0.8 (0.4-1.5)	1.04 (0.78 – 1.38)	0.780
IMATI	4.2 (2.2-8.4)	5.2 (2.7-8.6)	1.00 (0.95 – 1.05)	0.919

Data are mean ± standard deviation or median (IQR), or number (%). *p-value < 0.05 indicates statistical significance. Abbreviations: AST, aspartate aminotransferase; ALT, alanine aminotransferase; CRP, C-reactive protein; PNI, prognostic nutritional index; GNRI, geriatric nutritional risk index; HR, hazard ratio; CI, confidence interval; SMI, skeletal muscle index; SATI, subcutaneous adipose tissue index; VATI, visceral adipose tissue index; VSR, visceral-to-subcutaneous fat ratio; IMATI, intramuscular adipose tissue index.

Supplement Table S7. Diagnostic performance of nutritional and CT-based body composition parameters in predicting mortality.

**Variables**	**AUC (95% CI)**	**Sensitivity**	**Specificity**	**Cut-off value**	***p*-value**
PNI	0.70 (0.53–0.87)	77.8%	63.3%	≤ 47.3	0.020*
GNRI	0.76 (0.62–0.90)	78.2%	77.4%	≤ 94.5	<0.001*
Before chemotherapy					
L3					
SMI	0.72 (0.57–0.88)	78.6%	65.0%	≤ 44.9	<0.001*
SATI	0.51 (0.33–0.70)	79.2%	40.0%	≤ 75.2	0.935
VATI	0.58 (0.37–0.76)	79.2%	46.7%	≤ 82.2	0.385
VSR	0.56 (0.39–0.73)	45.8%	73.3%	≤ 0.9	0.442
IMATI	0.53 (0.36–0.69)	70.8%	53.3%	≥ 4.6	0.683
T12					
SMI	0.66 (0.48–0.85)	75.8%	50.0%	≤ 43.9	0.248
SATI	0.57 (0.37–0.77)	83.3%	46.7%	≤ 60.2	0.487
VATI	0.61 (0.44–0.77)	73.1%	43.3%	≤ 73.5	0.183
VSR	0.61 (0.46–0.76)	47.2%	80.0%	≤ 0.9	0.164
IMATI	0.61 (0.42–0.81)	71.7%	43.3%	≥ 9.4	0.145
Change after chemotherapy					
ΔL3					
SMI	0.85 (0.74–0.94)	82.9%	79.7%	≤ -10.4%	<0.001*
SATI	0.61 (0.45–0.75)	62.5%	66.7%	≤ -7.9%	0.175
VATI	0.60 (0.47–0.74)	43.1%	86.7%	≤ -24.9%	0.177
VSR	0.59 (0.44–0.74)	43.1%	86.7%	≤ -18.5%	0.265
IMATI	0.71 (0.59-0.82)	72.1%	74.6%	≥ +12.8%	0.023*
ΔT12					
SMI	0.70 (0.56-0.81)	72.4%	68.2%	≤ -7.8%	0.028*
SATI	0.52 (0.39–0.64)	40.3%	93.3%	≤ -11.2%	0.754
VATI	0.63 (0.49–0.76)	44.4%	86.7%	≥ -25.4%	0.083
VSR	0.62 (0.48–0.76)	55.6%	80.0%	≥ -14.1%	0.090
IMATI	0.68 (0.54-0.79)	69.2%	70.8%	≥ +18.4%	0.032*

Data are presented as area under the curve, with corresponding 95% confidence intervals (CI), sensitivity, specificity, and optimal cut-off values. Optimal cut-off values were determined using the Youden index. **p*-value < 0.05 indicates statistical significance. Abbreviations: PNI, Prognostic Nutritional Index; GNRI, Geriatric Nutritional Risk Index; SMI, skeletal muscle index; SATI, subcutaneous adipose tissue index; VATI, visceral adipose tissue index; VSR, visceral-to-subcutaneous fat ratio; IMATI, intramuscular adipose tissue index; Δ, post-chemotherapy change in parameter value.
